# Quantitative and semi-quantitative CT assessments of lung lesion burden in COVID-19 pneumonia

**DOI:** 10.1038/s41598-021-84561-7

**Published:** 2021-03-04

**Authors:** Xiaojun Guan, Liding Yao, Yanbin Tan, Zhujing Shen, Hanpeng Zheng, Haisheng Zhou, Yuantong Gao, Yongchou Li, Wenbin Ji, Huangqi Zhang, Jun Wang, Minming Zhang, Xiaojun Xu

**Affiliations:** 1 Department of Radiology, The Second Affiliated Hospital, Zhejiang University School of Medicine, No.88 Jiefang Road, Shangcheng District, Hangzhou, 31009 China; 2 Department of Radiology, Yueqing People’s Hospital, Yueqing, Wenzhou, Zhejiang China; 3 Department of Radiology, The Third Affiliated Hospital and Ruian People’s Hospital of Wenzhou Medical University, Ruian, Zhejiang China; 4 Department of Radiology, Taizhou Hospital of Zhejiang Province, Taizhou, Zhejiang China; 5 School of Biomedical Engineering, Shanghai Jiao Tong University, Shanghai, China

**Keywords:** Diagnostic markers, Respiratory tract diseases, Medical imaging, Radiography

## Abstract

This study aimed to clarify and provide clinical evidence for which computed tomography (CT) assessment method can more appropriately reflect lung lesion burden of the COVID-19 pneumonia. A total of 244 COVID-19 patients were recruited from three local hospitals. All the patients were assigned to mild, common and severe types. Semi-quantitative assessment methods, e.g., lobar-, segmental-based CT scores and opacity-weighted score, and quantitative assessment method, i.e., lesion volume quantification, were applied to quantify the lung lesions. All four assessment methods had high inter-rater agreements. At the group level, the lesion load in severe type patients was consistently observed to be significantly higher than that in common type in the applications of four assessment methods (all the p < 0.001). In discriminating severe from common patients at the individual level, results for lobe-based, segment-based and opacity-weighted assessments had high true positives while the quantitative lesion volume had high true negatives. In conclusion, both semi-quantitative and quantitative methods have excellent repeatability in measuring inflammatory lesions, and can well distinguish between common type and severe type patients. Lobe-based CT score is fast, readily clinically available, and has a high sensitivity in identifying severe type patients. It is suggested to be a prioritized method for assessing the burden of lung lesions in COVID-19 patients.

## Introduction

The coronavirus disease 2019 (COVID-19), caused by severe acute respiratory syndrome coronavirus 2 (SARS-CoV-2), is an ongoing pandemic^1^ that greatly influence human life and social economy. By June 30, 2020, more than 10 million cases of COVID-19 have been reported in over 200 countries and territories. A lot of affected patients develop pneumonia (called novel coronavirus pneumonia, NCP), and some of them will progress to severe respiratory failure, which can lead to death^2^. Thus, clinically assessing the severity of NCP is a critical way to better understand the disease status. Chest computed tomography (CT) is an important and useful technique in the diagnosis and evaluation of lung diseases including pneumonia. Thus, for the COVID-19 patients with NCP, chest CT may play a critical role in case ascertainment, disease monitoring and management.

The most common CT manifestations of NCP are multiple patchy areas of ground glass opacity and consolidation, which are predominately located in the periphery of the lungs^3,4^. Many studies have demonstrated that lung lesions in NCP patients varied from the stages of the disease^5,6^. The disease progression is found to be associated with both increased numbers and sizes of ground glass opacity in combination with consolidative opacities. In the process of remission, the extent and density of lung lesions significantly reduced^7,8^. Therefore, the extent of lung tissue involvement, that is the burden of lung lesion, could largely reflect the severity of the NCP disease. Evaluating the lung lesion burden of NCP patients will be helpful in determining the severity of the disease, and thus to stratify the patients’ management and treatment.

Several evaluation methods have been applied to the assessment of lung lesion burden in patients with NCP. The most commonly used is a semi-quantitative evaluation method called CT score or severity score. The lesion opacifications and size in each lobe^9,10^ or segment^11,12^ of the lung are estimated and counted. A score is assigned according to the percentage of involvement in each lobe or segment. All lobar or segmental scores are summed to calculate the overall score as a metric for the severity of opacifications. More recently, lesion volume measurement, as a quantitative method, has become a promising approach to quantifying the disease loads for COVID-19, and has been shown to have the ability to predict the disease progression^13,14^. However, although researchers have claimed that these semi-quantitative or quantitative methods can be employed to evaluate the severity of lung inflammation in NCP patients, few studies have compared the effectiveness between these methods directly. Therefore, it remains largely unknown that whether these different methods would provide more details than the one another.

In the present study, we employed four different measurements, including semi-quantitative and quantitative methods, to evaluate the lung lesion burden in NCP patients that were recruited from three hospitals in Zhejiang Province, China. The purpose of this study is to clarify the effectiveness of different assessment methods in reflecting the severity of the NCP, and then provide evidence for the selection of clinically applicable methods.

## Methods

### Patients

A total of 244 patients were retrospectively recruited from three local hospitals in Zhejiang Province (81 patients from Site 1; 66 patients from Site 2; 97 patients from Site 3). This study was approved by the Medical Ethic Committee of Yueqing People's Hospital, Ruian People's Hospital and Taizhou Hospital of Zhejiang Province, and was also reviewed by the Medical Ethic Committee of the Second Affiliated Hospital of Zhejiang University School of Medicine. The written informed consent from the patients was waived, which was approved by the above institutes. All the methods were carried out in accordance with relevant guidelines and regulations.

For all the patients, the diagnosis of COVID-19 was confirmed by performing the real-time reverse transcriptase polymerase chain-reaction (RT-PCR) on nasal and pharyngeal swab specimens. Based on the Diagnosis and Treatment Plan of COVID-19 issued by the National Health Commission of China^15^, COVID-19 patients were divided into four types: (1) Mild type: mild clinical symptoms without pneumonia in imaging; (2) common type: fever, respiratory tract and other symptoms with pneumonia in imaging; (3) severe type: respiratory distress, respiratory rate ≥ 30 times/min; in resting state, oxygen saturation ≤ 93%; PaO_2_/FiO_2_ ≤ 300 mmHg; (4) critical type: respiratory failure requiring mechanical ventilation, shock and other organ failure requiring ICU monitoring and treatment. According to the above criteria, 31 of the 244 patients were assigned to mild type, 175 patients were assigned to common types, 38 patients were assigned to severe type, and there were no critical type patients (Table 1).

**Table 1:** The demographic and clinical information of the multi-center database of COVID-19.

	Site 1	Site 2	Site 3
Number	81	66	97
Age (years)	44.4 ± 15.4	49.4 ± 13.4	45.8 ± 13.3
Gender (male/female)	42/39	33/33	52/45
Clinical type (mild/common/severe)	19/55/7	3/41/22	9/79/9

### CT scanning

All patients underwent chest CT scanning on admission. CT scans were obtained with the patients in the supine position, and scanning was performed at the end of clinical inspiration without intravenous contrast. The scanning range was from the apex to lung base. All the patients were scanned in one of three CT scanners (Somatom Perspective and Somatom Scope, Siemens Healthcare, Erlangen, Germany; uCT 530, United Imaging, Shanghai, China) with the following parameters: tube voltage, 100–120 kV; tube current, automatic tube current modulation or 175 mA; ref mAs, 70–130; pitch, 0.9–1.175; matrix, 512 × 512; and section thickness after reconstruction, 5.0 mm, with a sharp reconstruction kernel (B_SHARP_C) or a pulmonary B70s kernel.

### Image analysis

Since patients with mild type of COVID-19 have no lesions on the lung images, only 213 patients with common type and severe type were included in the subsequent CT image analysis. Lung windows with window level at − 500 HU and window width at 1500 HU were used to display CT images.

Two experienced radiologists (radiologist 1, with 20 years of diagnostic radiology experience, and radiologist 2, with 10 years of experience) independently reviewed chest CT images of all the patients. Three semi-quantitative CT scoring methods were used to access the severity of lung disease according to the following criteria: (1) Segmental based assessment: each of the 18 lung segmentations was visually evaluated using a system attributing scores of 0, 1, and 2 (0, no parenchymal opacification; 1, parenchymal opacification involvement less than 50%; 2, involvement more than 50%). The total score was the sum of the individual segmentation scores and ranged from 0 (no involvement) to 36 (maximum involvement)^12^. (2) Lobar based assessment: each of the 5 lung lobes was subjectively scored from 0 to 5 (0, no involvement; 1, involvement < 5%; 2, involvement 6–25%; 3, involvement 26–50%; 4, involvement 51–75%; 5, involvement > 75%). The total score was the sum of the individual lobar scores and ranged from 0 to 25^9^. (3) Opacity-weighted segmental assessment: based on the segmental assessment, we further added ground glass opacity and consolidation opacity, ranging from 1 to 2. The total score was the sum of the opacity-weighted individual segmental scores and ranged from 0 to 72.

**Figure 1:**
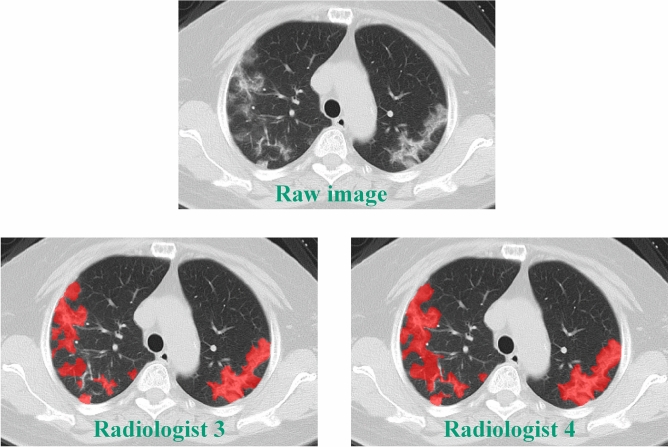
Manual Lesion segmentation by Radiologist 3 and Radiologist 4. This is an example of lesion segmentation in a 68 year old female patient from Site 1. She was assigned to the common type of COVID-19 patients. The Dice similarity coefficient of the segmented inflammatory lesions between Radiologist 3 and Radiologist 4 is 0.812.

Meanwhile, the quantitative assessment of lung lesions was performed by one of two radiologists (radiologist 3, with 6 years of diagnostic radiology experience; radiologist 4, with 8 years of experience). ITK-SNAP (http://www.itksnap.org) was used to manually segment the lung lesions on each slice of CT image along the edge of parenchymal opacification. The lung lesion volume of each patient was the sum of the lesion volume on each CT slice image. In order to confirm the robustness of manual segmentation (Fig. 1), 10 patients were randomly picked out from the whole database and were segmented respectively by these two radiologists.

### Statistical analysis

The age distribution among each type of patient was compared by using Analysis of Variance (ANOVA), while Chi-square was used to compare the gender distribution. Intra-class correlation coefficient (ICC) analysis was conducted to test the assessment reliability of semi-quantitative CT scoring between two radiologists, where ICC < 0.50 means poor reliability, between 0.50 and 0.75 indicates moderate reliability, between 0.75 and 0.90 means good reliability and > 0.90 means excellent reliability^16^. Dice similarity coefficient was calculated for testing the reliability of lesion segmentation between two radiologists, where the value of > 0.70 indicates an excellent agreement^17,18^.

In the subsequent inter-group comparisons, a general linear model was performed to detect the differences of CT score and lesion volume between common type and severe type patients with age and gender as covariates. At the individual level, receiver operating characteristic curve (ROC) was computed to test the discriminative efficacy of different methods between common type and severe type patients. Sensitivity, specificity and area under the curve (AUC) were calculated, and the Youden index was used to determine the best discriminative result. P < 0.05 was regarded as statistically significant.

In order to clarify the effect of age on the disease severity of NCP patients, we further stratified patients into six groups (Group 1, 21–30 years old; Group 2, 31–40 years old; Group 3, 41–50 years old; Group 4, 51–60 years old; Group 5, 61–70 years old; Group 6, 71–86 years old). ANOVA with Least Significant Difference adjustment was conducted to observe the inter-group lesion load differences that were assessed by the four approaches mentioned above. P < 0.05 was regarded as statistically significant.

## Results

### Demographic information

The COVID-19 patients with severe type were significantly older than the patients with mild type and common type (both p < 0.001). No significant difference in the gender distribution was observed (p = 0.280) (Table 2).

**Table 2:** The demographic information and lesion loads of each type of COVID-19 patients.

	Mild	Common	Severe	P value
Sample size	31	175	38	–
Age (years)	37.4 ± 19.8	46.6 ± 12.3	52.4 ± 13.5	< 0.001
Gender (male/female)	17/14	86/89	24/14	0.28
Lobar lesion assessment	–	5.1 ± 3.2	12.1 ± 4.2	1.6 × 10^–21^
Segmental lesion assessment	–	7.5 ± 5.5	17.9 ± 6.1	1.3 × 10^–18^
Opacity-weighted lesion assessment	–	9.0 ± 6.8	23.5 ± 10.0	1.6 × 10^–20^
Lesion volume (mm^3^)	–	9 × 10^4^ ± 1.1 × 10^5^	4.9 × 10^5^ ± 3.4 × 10^5^	3.1 × 10^–25^

### The reliability of semiquantitative methods

In the lobar lesion assessment, radiologist 1 and 2 achieved excellent agreement with a mean individual ICC of 0.919 (95% confidence interval, 0.877–0.944; p < 0.001). In the segmental lesion assessment, they also achieved excellent agreement with a mean individual ICC of 0.986 (95% confident interval, 0.979–0.990; p < 0.001) (Fig. 1 and Table 3). In the opacity-weighted lesion assessment, similarly excellent agreement with a mean individual ICC of 0.937 was achieved (95% confident interval, 0.910–0.955; p < 0.001). Therefore, these data demonstrated that the reliability of semi-quantitative methods was high, and in the following analysis we calculated the averaged CT severity score instead of using either score made by radiologist 1 or radiologist 2.

**Table 3:** The ICC results of lobar/segmental lesion assessments between Radiologist 1 and Radiologist 2.

	Radiologist 1	Radiologist 2	ICC	95% CI	P value
Lobar	6.0 ± 4.3	6.6 ± 4.6	0.919	0.877–0.944	< 0.001
Segmental	9.7 ± 7.1	9.0 ± 6.8	0.986	0.979–0.990	< 0.001
Opacity-weighted	12.1 ± 9.8	11.1 ± 9.0	0.937	0.910–0.955	< 0.001

*ICC* intraclass correlation coefficient, *CI* confident interval, *CI* confidence interval.

### The reliability of lesion segmentation

**Figure 2:**
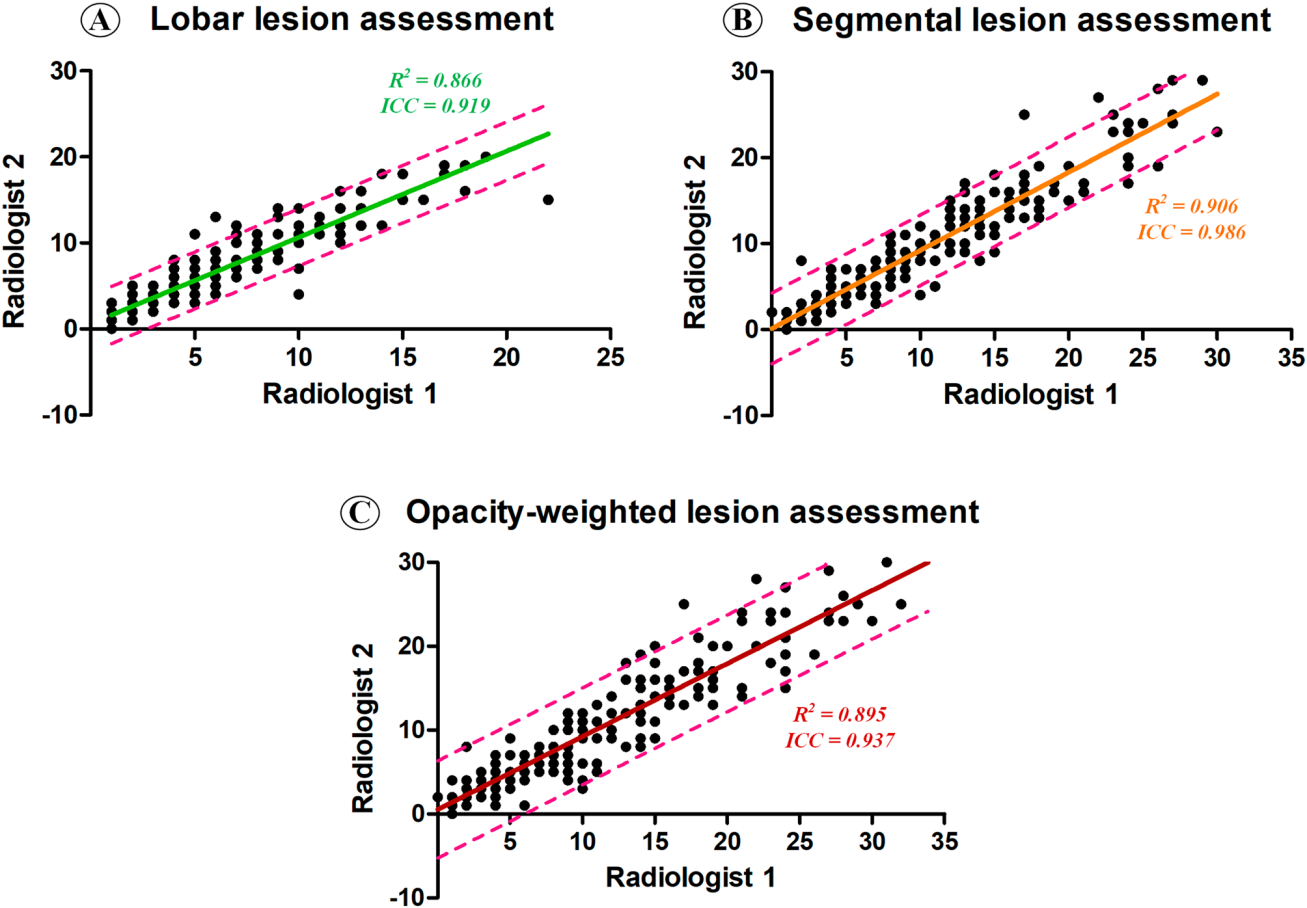
The correlation of semiquantitative lesion assessments between Radiologist 1 and Radiologist 2.

The Dice similarity coefficient was 0.815 ± 0.032, ranging from 0.758 to 0.857, indicating that the lesion segmentation was highly robust across the two radiologists (Fig. 2).

### The discriminative ability to identify different types of COVID-19 patients

At the group level (Table 2), compared to common type, severe type patients had significantly higher lesion loads measured by lobar lesion assessment (p < 0.001), segmental lesion assessment (p < 0.001), opacity-weighted lesion assessment (p < 0.001), and lesion volume quantification (p < 0.001).

**Figure 3:**
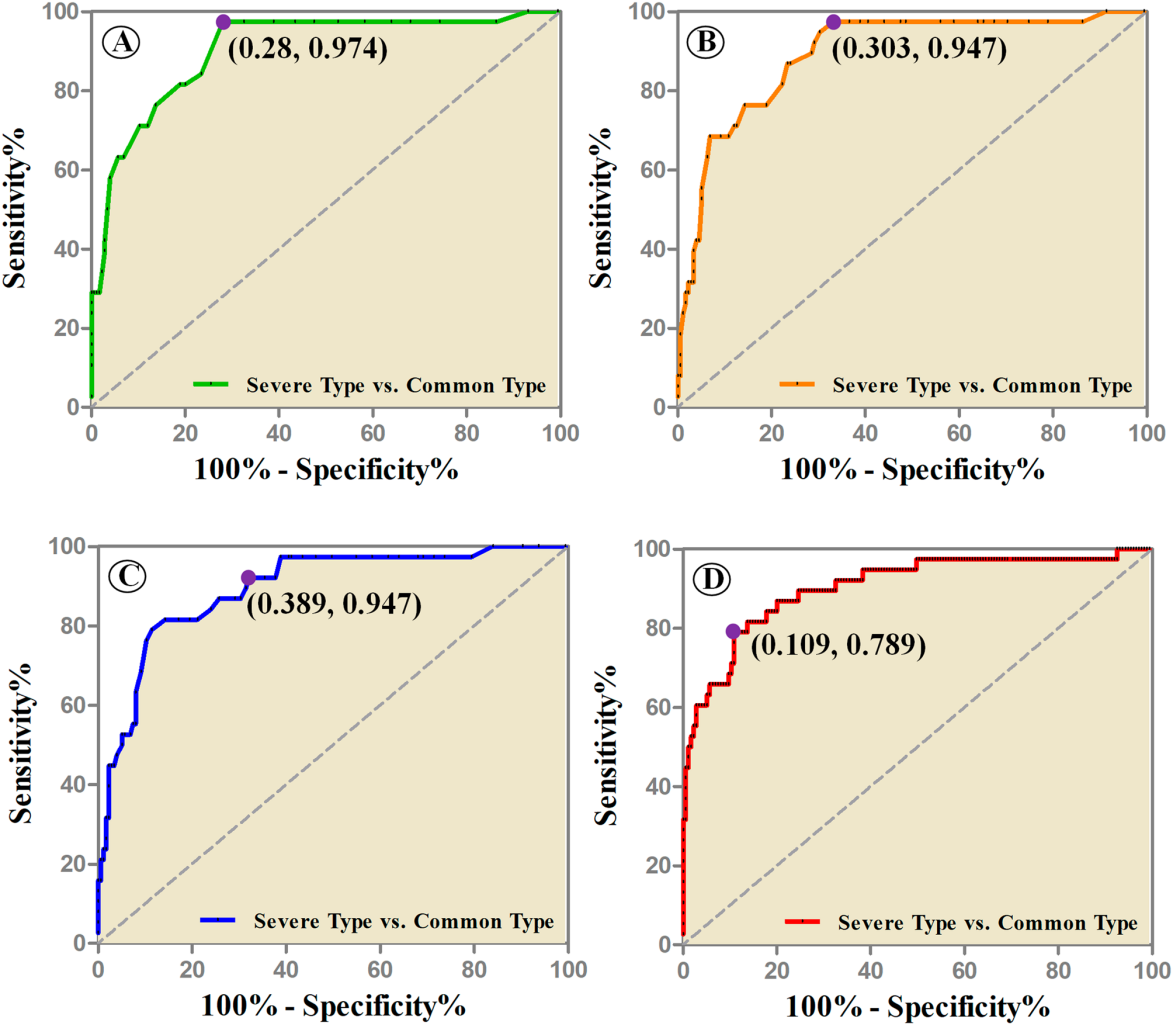
The discriminative efficacy (ROC analysis) of lesion assessments, measured by lobar lesion assessment (**A**), segmental lesion assessment (**B**), opacity-weighted lesion assessment (**C**), and lesion volume quantification (**D**), between severe type and common type patients with COVID-19.

At the individual level (Fig. 3), we observed that, (1) a lobar disease severity score of 6.75 could discriminate between the two types of COVID-19 patients with AUC, sensitivity and specificity values of 0.904, 97.4% and 72.0%, respectively; (2) the segmental disease severity score of 9.75 could discriminate between the two types of COVID-19 patients with AUC, sensitivity and specificity values of 0.891, 94.7% and 69.7%, respectively; (3) a opacity-weighted disease severity score of 9.75 could discriminate the two types of COVID-19 patients with AUC, sensitivity and specificity values of 0.894, 94.7% and 61.1%, respectively; and (4) a lesion volume of 227,064.75 mm^3^ could discriminate between the two types of COVID-19 patients with the AUC, sensitivity and specificity values of 0.903, 78.9%, and 89.1%, respectively.

Thus, the semiquantitative measurements, e.g., lobar disease severity, segmental disease severity, and opacity-weighted disease severity, had high true positives and an excellent ability to identify severe type patients, while the quantitative measurement, lesion volume quantification, had high true negatives, indicating a good screening of common type patients.

### The alterations of lesion load among patients with different ages

**Figure 4:**
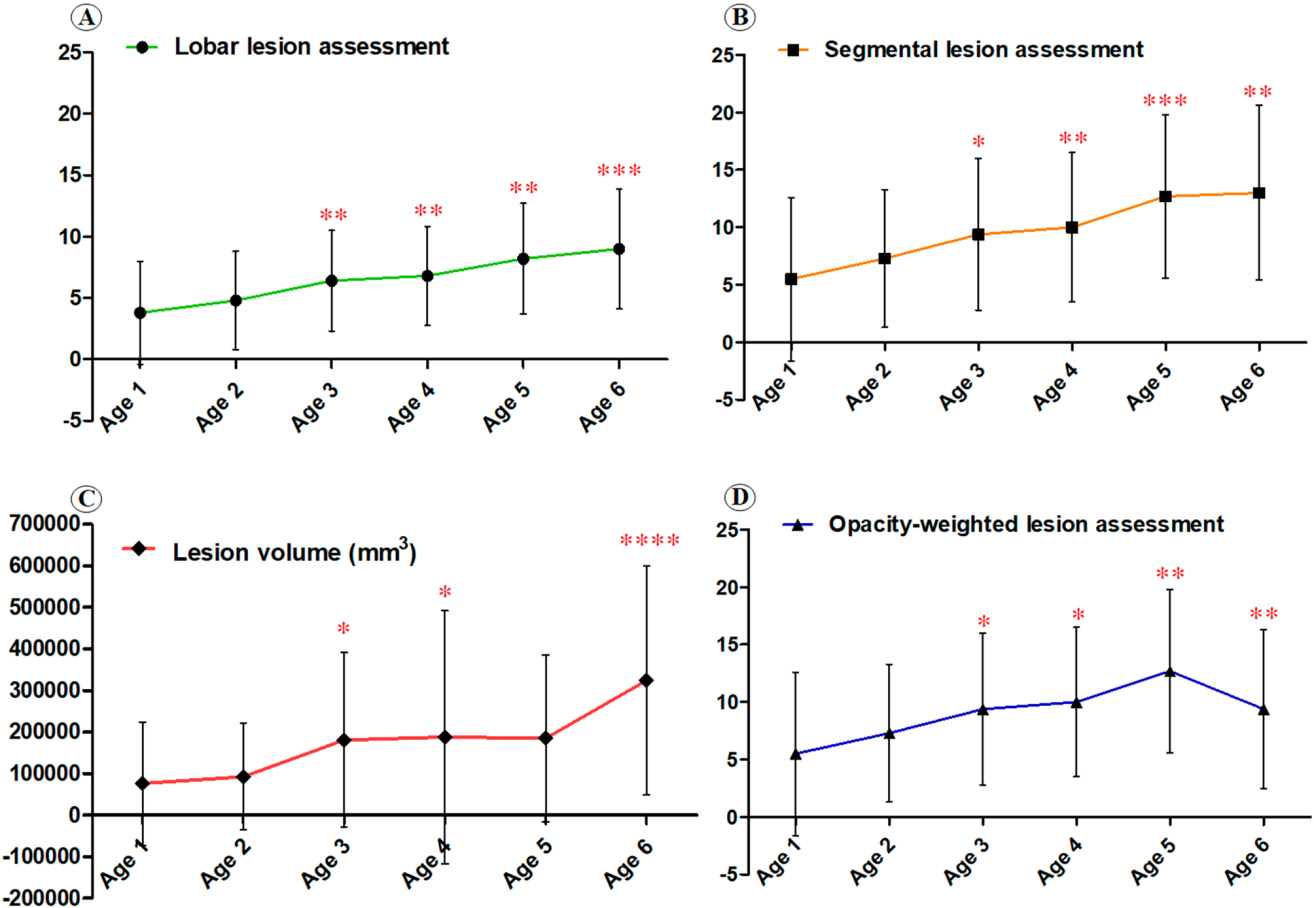
The inter-group differences among COVID-19 patients with different ages. The number of asterisk(s) above each group means the number of inter-group differences showing statistical significance when comparing the lesion load in this group with that in the other group(s) who were younger than them. In each comparison, the asterisk means p < 0.05 with Least Significant Difference adjustment.

213 patients with COVID-19 were assigned to six groups according to ascending ages (Fig. 4). In the lobar lesion assessment, patients in the Age 6 group had significantly higher lesion load than patients in the Age 3 (p = 0.032), Age 2 (p = 0.001) and Age 1 (p < 0.001) groups; patients the in Age 3, Age 4 and Age 5 groups had significantly higher lesion load than patients in the Age 2 (p = 0.050, 0.022 and 0.001, respectively) and Age 1 (p = 0.021, 0.011 and 0.001, respectively) groups.

In the segmental lesion assessment, patients in Age 4, Age 5 and Age 6 groups had significantly higher lesion load than patients in the Age 2 (p = 0.046, 0.001 and 0.004, respectively) and Age 1 (p = 0.015, 0.001 and 0.001, respectively) groups. Moreover, patients in the Age 5 group had additionally higher lesion load than the patients in the Age 3 group (p = 0.040), while patients in the Age 3 group had more lesion load than the patients in the Age 1 group (p = 0.033).

In the opacity-weighted lesion assessment, patients in the Age 3–6 had significantly higher lesion load than patients in the Age 1 (p = 0.050, 0.033, 0.009, and 0.013) while patients in the Age 5 and Age 6 had significantly higher lesion load than patients in the Age 2 (p = 0.014 and 0.024).

In the lesion volume quantification, patients in the Age 6 group had larger lesion volume than patients in the Age 1–4 groups (p = 0.002, 0.001, 0.027 and 0.038, respectively). Besides, patients in the Age 3 and Age 4 groups were found to have larger lesion volume than patients in the Age 2 group (p = 0.045 and 0.037).

In summary, all four methods consistently demonstrated that COVID-19 patients with older ages were more susceptible to suffer from severe disease involvement.

## Discussion

Recently, the Fleischner Society^19^, European Society of Radiology^20^, European Society of Thoracic Imaging^21^, and British Society of Thoracic Imaging^22^ have successively issued expert consensus or statements on the role of imaging in the COVID-19 epidemic. They clarified the essential role of chest CT in the evaluation of disease severity in NCP patients, which may help to predict patient outcome and to facilitate medical triage and associated decisions regarding disposition, infection control, and clinical management. Thus, the investigation of agreement among different methods of assessing lung lesion burden became very urgent, which would be of value in improving the consensus of radiologist clinical activity and facilitating research standardization, since potential discrepancies have not been disclosed.

In the present study, we employed three semi-quantitative methods based on lobar CT score, segmental CT score and opacity-weighted CT score respectively, and a quantitative method based on lesion volume quantification to assess the severity of lung lesions in COVID-19 patients. First of all, high agreement between radiologists (ICC and DICE similarity coefficient) for these four assessment methods in measuring lesion loads were demonstrated, indicating that all four evaluation results are highly repeatable. Then, we found that there were significant differences between the common type and severe type patients in the lobar CT score, segmental CT score, opacity-weighted CT score and lesion volume, suggesting that all four methods can distinguish the common type from severe type at the group level. Lastly, we found that, at the individual level, although all four methods had shown comparable AUC values, the results were different in that the semi-quantitative assessments had high true positives when discriminating severe type COVID-19 patients from common type, while the quantitative lesion volume had high true negatives.

The semi-quantitative method for lung disease originated from the study of SARS more than 10 years ago^9,10^. Recently, many researchers have adopted these methods to assess the severity of lung lesions in NCP patients. They found that severity score based on visual inspection of the number and size involved within each lobe or segment correlated well with disease stages or durations^5,6^. In this study, we observed these four different scoring methods had similar efficacy in distinguishing common type from severe type patients, which provided sufficient and direct evidence that the research outcomes from the previous researches^5,6^ that employed either CT scoring method were highly comparable. Additionally, since scoring based on lung lobes is more convenient than that based on lung segments, we suggested that the lobar based assessment method may be more suitable for clinical application.

Semi-quantitative methods will inevitably encounter the ceiling effect on assessing lung lesions, leading to potentially biased results; however, no relevant evidence have been clarified. Recently, lesion volume measurement has become a promising approach for absolutely quantifying the disease burden for NCP, and has been shown to have the ability to predict the disease progression^13^. In theory, the measurement of lung lesion volume can accurately reflect the burden of the lesion and may be more helpful in distinguishing patients with different clinical severities. However, it was surprising that the quantitative method based on lesion volume measurement in this study did not show any advantage in distinguishing the severity of the disease. Compared with semi-quantitative methods, quantitative evaluation had higher specificity but lower sensitivity. We would like to argue that, since patients with severe NCP have high risk of disease progression and mortality, for the purpose of improving the prognosis, timely identifying patients with severe type should be prioritized, giving enough time for clinical interventions; therefore, the semi-quantitative method with relatively high sensitivity is more qualified. Moreover, the time–cost of lesion assessment is another important consideration. Very recently, Mei et al. proposed an artificial intelligence system to help rapidly diagnose COVID-19 patients by integrating chest CT findings, clinical symptoms, exposure history and laboratory testing^23^, which gives an innovative and timesaving application of deep learning to public emergent events. Likewise, a deep learning framework, specifically for automatic segmentation of inflammatory lesion, should have a promising potential to maximally reduce labor time. However, such artificial intelligence model is still undergoing development and only used locally^24,25^, precise volume quantification is largely dependent on manual segmentation currently, which is consuming precious time and energy, making it difficult to be widely clinically applied. Taken together, these data disclosed that semiquantitative method, e.g., the lobar lesion assessment, is more optimal measurement for urgent usefulness in identifying severe COVID-19 patients with high diagnostic sensitivity and low time–cost.

As presented in the demographic information, the patients with severe type were older than the patients with mild type and common type. We searched for relevant evidence for such clinical status and further revealed that, the lung lesion loads in the old patients, measured by all the four methods, were higher than that in the young patients with an ascending tendency, indicating older patients may be more susceptible to be severely affected by the SARS-CoV-2 infection. Previous studies have reported that the elderly have a higher mortality rate among COVID-19 patients, and the elderly account for a larger proportion of deaths^2,26^. This phenomenon may be related to poor immunity and complicated underlying diseases (such as diabetes, coronary heart disease, etc.) in the elderly^2,27^, which was also demonstrated during the SARS epidemic^28^. Therefore, we speculated that the higher lung lesion load in the older patients can be one of the important predictors reflecting the serious underlying inflammatory response leading to a poor prognosis in this special population. Of note, the time-saving methods, like the lobar lesion assessment, also performed well in the investigations compared with the time-costing method, volume quantification.

There were several limitations in this study. First, although patients from three hospitals were recruited, there was a lack of critical type patients among them. Therefore, it was impossible to assess whether the above methods could distinguish critical patients from other types. Second, because this was a retrospective study and a lot of patients were recruited from emergency clinic, thin-slice CT images were not commonly collected. Therefore, we used 5 mm-thick CT images for lesion interpretation and segmentation. Bias may be induced when comparing these results with that of thin-slice CT images. Third, this study only used the cross-sectional data and images of patients, and the value of the above methods in assessing longitudinal changes in lung lesion burden is not clear.

## Conclusions

Both semi-quantitative and quantitative methods have excellent repeatability in measuring inflammatory lesions, and can well distinguish between common type and severe type patients. Lobar-based CT score with high sensitivity in identifying severe type patients, low time consumption and readily clinical availability is suggested to be a prioritized method for assessing the burden of lung lesions in COVID-19 patients.

## Data Availability

Anonymized data can be made available upon reasonable request to the corresponding author.
